# Cellular Responses to Platinum-Based Anticancer Drugs and UVC: Role of p53 and Implications for Cancer Therapy

**DOI:** 10.3390/ijms21165766

**Published:** 2020-08-11

**Authors:** David Murray, Razmik Mirzayans

**Affiliations:** Department of Oncology, Cross Cancer Institute, University of Alberta, Edmonton, AB T6G 1Z2, Canada; razmik.mirzayans@ahs.ca

**Keywords:** cisplatin and analogs, UVC, cancer, apoptosis, therapy-induced cell senescence, polyploid giant cells, treatment failure

## Abstract

Chemotherapy is intended to induce cancer cell death through apoptosis and other avenues. Unfortunately, as discussed in this article, moderate doses of genotoxic drugs such as cisplatin typical of those achieved in the clinic often invoke a cytostatic/dormancy rather than cytotoxic/apoptosis response in solid tumour-derived cell lines. This is commonly manifested by an extended apoptotic threshold, with extensive apoptosis only being seen after very high/supralethal doses of such agents. The dormancy response can be associated with senescence-like features, polyploidy and/or multinucleation, depending in part on the p53 status of the cells. In most solid tumour-derived cells, dormancy represents a long-term survival mechanism, ultimately contributing to disease recurrence. This review highlights the nonlinearity of key aspects of the molecular and cellular responses to bulky DNA lesions in human cells treated with chemotherapeutic drugs (e.g., cisplatin) or ultraviolet light-C (a widely used tool for unraveling details of the DNA damage-response) as a function of the level of genotoxic stress. Such data highlight the growing realization that targeting dormant cancer cells, which frequently emerge following conventional anticancer treatments, may represent a novel strategy to prevent or, at least, significantly suppress cancer recurrence.

## 1. Introduction: Cellular and Molecular Responses to Cisplatin and UVC

Many preclinical anticancer drug discovery/optimization strategies are based on the assumption that there is some degree of dose-linearity in the various cellular and molecular responses to cytotoxic drugs that invoke genotoxic/oxidative stress. When investigating the mechanism of action of such agents or of the modulation thereof, e.g., using pharmacological adjuvants in an attempt to improve therapeutic index or to overcome drug resistance, researchers are often faced with an immediate problem. This relates to the selection of the dose level of the cytotoxic agent to use, e.g., for high-throughput automated screening studies that use dye-based assays to monitor specific endpoints such as apoptosis, mitochondrial metabolism/dysfunction and viability (cell membrane integrity). Often, a high/supralethal dosage is selected with the expectation of a better signal-to-noise ratio. Such decisions often turn out to be justified; sometimes, they can be misinformative [1]. This is typically because some key feature of the dose-response curve displays nonlinearity. As such, the behavior of cells experiencing very high/supralethal levels of stress may not be informative for the cellular/molecular responses that occur at lower, biologically/clinically- relevant levels of injury where active cell survival-death decisions operate.

The biological response of human cancer cells to anticancer drugs has often been based on the use of the colony-forming (CF) assay. Such assays provide a readout of the clonogenic potential of the cells and integrate all modes of cytotoxic and cytostatic responses that operate within a cell culture over a span typically of approximately 2 weeks. However, it is now recognized that some of the molecular/cellular responses that underlie the loss of CF ability can exhibit distinctly nonlinear and sometimes even discontinuous dose-response curves (e.g., [2,3]). For example, as will be discussed below, many human cell lines, both normal and cancerous, display a distinct dose threshold for DNA damage-induced apoptosis.

The primary focus of this article is on the cellular and molecular effects of the electrophilic anticancer drug cisplatin and its analogs. Cisplatin is widely used in the treatment of many solid cancers, including those of the lung, cervix, testes, ovary, breast, bladder, head and neck, esophagus and brain, as well as mesothelioma, neuroblastoma, sarcomas, and hematological cancers such as leukemias and lymphomas [4,5,6,7]. Almost all of the major reviews on this topic (e.g., [4,5,6,7,8,9,10]) concur that the therapeutic effects of cisplatin against solid tumour-derived cells are primarily a result drug-induced DNA damage, although non-DNA targets may also contribute (e.g., [5,7]). Cisplatin-induced damage to DNA includes mono-adducts, intra-strand crosslinks and interstrand crosslinks. These same reviews further suggest that these DNA lesions lead to inhibition of DNA replication and activation of the DNA damage response (DDR) network, resulting either in cell survival or in cell death, primarily via the intrinsic/mitochondrial apoptotic pathway, although cisplatin [11,12] and indeed ultraviolet light-C (UVC) [13], which will be discussed below, can also activate apoptosis via the endoplasmic reticulum (ER) stress/unfolded protein response (UPR) pathway. Alternative cell fates, such as necrosis and cytostatic/dormancy responses characterized by a sustained proliferation arrest (which will be addressed in Section 4.2), do receive occasional mention [14].

We will also consider the effects of a second DNA-damaging agent, UVC, which not only shares some features with cisplatin but also exhibits notable differences. UVC, while having little direct relevance to human cancer because it is largely absorbed by the atmosphere, has proven to be an important research tool for unraveling details of the DDR. It has been particularly useful in this context because it is a physical (rather than chemical) agent and can therefore be delivered as a short pulse, such that the early DNA-damaging events can be interrogated without the complication of their continuing modification by cellular response pathways. Indeed, the initial yield of DNA photoproducts such as cyclobutane dimers and (6–4) photoproducts is linear over a broad range of UVC doses [15]. Importantly, the levels of initial DNA damage caused by UVC can be reasonably reproduced in different laboratories for a given cell type, facilitating inter-study comparisons.

With electrophilic drugs such as cisplatin and its analogs, such inter-study congruence is not necessarily the norm. Here, the initial level of cellular damage is subject to a number of factors that can vary greatly, such as: uptake of the drug by transporters such as the copper-transporting CTR1/2 or the organic cation transporters (OCTs); export of the drug from the cells by the copper-transporting P-type ATPases ATP7A/B or by the multidrug resistance-associated proteins 1 and 2 (MRP1/2, which export glutathione (GSH)-cisplatin conjugates) or multidrug resistance protein 1 (MDR1)/p-glycoprotein; and binding/inactivation of the electrophilic drug by expendable nucleophiles such as GSH, either directly or catalyzed by one of the GSH-S-transferase (GST) family of detoxifying enzymes [10,16,17,18]. Other anti-electrophile/antioxidant systems that vary with cell type and culture conditions include the cysteine-rich metallothioneins and the components of the pentose phosphate cycle-thioredoxin reductase-thioredoxin (PPC-TrxR-Trx) pathway which also facilitates the activation of pro-survival transcription factors such as NF-κB and AP-1 as well as of p53 [18,19]. The nuclear erythroid-like factor 2 (Nrf2) transcription factor, which is induced by oxidative stress and electrophilic/reactive oxygen species (ROS)-generating drugs, including cisplatin [20], can transcriptionally activate many of these anti-electrophile/antioxidant genes/proteins that in turn abrogate the cellular stress levels. Differences in pH (which depend in part on PPC activity via the regulation of glycolysis) and on drug exposure times can also have a profound impact on the loss of cellular CF ability [18]. Dissecting the molecular responses to cisplatin is further compounded by the delayed kinetics of DNA interstrand crosslink evolution, which can continue for many hours after the drug exposure [21].

A caveat here is that, while both cisplatin and UVC primarily induce bulky DNA lesions (DNA photoproducts and mono-adducts/intra-strand crosslinks, respectively) that are processed by the nucleotide excision repair (NER) pathway, cisplatin also induces a low (but potentially important) level of interstrand crosslinks. Interstrand crosslinks bring a different set of DNA-repair factors into play, such as the homologous recombination repair, translesion synthesis and the Fanconi anemia (FA) pathways [22,23], so the stress signaling response after cisplatin will be broader than that invoked by UVC. The mismatch repair pathway is also involved in processing DNA lesions induced by both cisplatin [24] and UVC [25].

**Figure 1:**
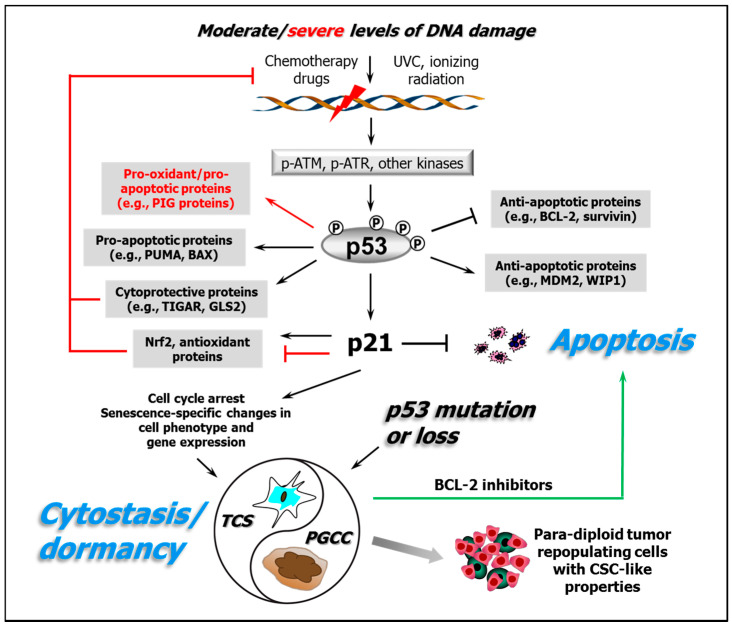
A partial schematic of the DNA damage response network illustrating the importance of both the positive and negative regulatory activities of p53 and its downstream target p21 on proteins involved in cytoprotective/antioxidant and cytotoxic/apoptotic pathways. The arrows indicate stimulation, and the T-shaped lines indicate inhibition; the black lines/text refer to responses seen following exposure of cells to moderate concentrations/doses of DNA-damaging agents typical of those where the progressive loss of colony-forming ability is seen; and the red lines/text refer to responses to high/supralethal exposures. The green arrow represents the strategy discussed in Section 5 whereby pharmacological inhibitors of antiapoptotic proteins such as BCL-2 and BCL-XL might be clinically useful for transitioning chemotherapy-induced dormant cancer cells (which have the potential to cause tumour recurrence/metastasis via the generation of para-diploid cancer stem cell (CSC)-like tumour repopulating cells) into an apoptotic cell-death pathway from which they will not return. TCS, therapy-induced cell senescence; PGCC, polyploid giant cancer cell. The protein abbreviations are provided in the abbreviation list.

Here, we will consider aspects of the shapes of some of the dose-response curves for cellular responses to cisplatin and UVC and for activation of the underlying molecular pathways that regulate them as well as the implications of these findings for studying mechanisms of anticancer activity and their modification. Figure 1 illustrates key responses triggered by DNA-damaging agents in human cancer cells with differing p53 status as discussed below.

## 2. Roles of p53 in Cellular Responses to Genotoxic/Oxidative Stress

The wild-type (*WT*) p53 protein coordinates many aspects of the cellular/molecular response to DNA damage [3,26,27,28,29,30,31,32]. Under non-stress conditions, p53 is kept at low levels primarily by the p53 regulators MDM2 and MDM4 (murine double minute 2 and 4 homologues) [33,34]. Following DNA damage, p53 is subject to various posttranslational modifications, notably phosphorylation either directly by the activated ATM (ataxia telangiectasia-mutated) and/or ATR (ataxia telangiectasia and Rad3-related) serine-threonine kinases or indirectly by their substrate checkpoint kinases Chk2 and Chk1. After cisplatin or UVC exposure, p53 is phosphorylated preferentially by ATR, primarily in response to persistent single-stranded DNA regions, notably those generated by DNA replication stress and at certain DNA repair intermediates as well as by DNA double-strand breaks (DSBs); ATM is preferentially activated by DSBs or chromatin disruption [29,35,36]. Other key targets of activated ATR-Chk1 and ATM-Chk2 include MDM2 and MDM4, phosphorylation of which disrupts the p53-MDM2/4 interactions. While ATR and ATM phosphorylate many of the same substrates [35], they may also have unique targets. For example, the ATR-Chk1 pathway engages in crosstalk with the FA pathway which is involved in DNA interstrand crosslink repair and thus in the cellular response to cisplatin and its analogs [22,23,29].

As a result of these collective events, p53 undergoes transient stabilization and activation followed by its relocation to the nucleus, where it triggers the transcription of numerous target genes [3,37]. These encode proteins involved in activities such as the early activation of various DNA repair pathways and transient cell cycle checkpoints as well in later events, e.g., proapoptotic genes/proteins such as BAX (BCL-2-associated X) and PUMA (p53 upregulated modulator of apoptosis). p53 can also favor apoptosis by downregulating antiapoptotic proteins such as survivin and BCL-2 (B-cell lymphoma 2) [38,39]. Another p53-regulated gene, *CDKN1A*, encodes the p21^WAF1^ (henceforth, p21) protein, which plays key roles in both early and late responses to DNA damage, such as in the transient activation of cell cycle checkpoints and in the sustained growth arrest (cytostasis/dormancy) seen in many cell types [3,39,40]. p21 can also inhibit apoptosis via a number of mechanisms, including downregulation/inhibition of proapoptotic genes/proteins and upregulation of antiapoptotic genes/proteins [39].

Aspects of these ATR/ATM-mediated molecular responses to DNA damage induced by agents such as cisplatin and UVC are strongly dose- and time-dependent [41,42,43,44,45]. We will illustrate this here by reference to an early study by Latonen et al. [41]. These authors reported that human fibroblasts displayed increased levels of MDM2 and its association with p53 following a low dose exposure to UVC (10 J/m^2^) but not after a high dose (50 J/m^2^) exposure. Furthermore, the low-dose exposure resulted in a transient cell cycle checkpoint activation, whereas the high dose triggered an early apoptotic response within 12 h. Low-dose UVC caused a rapid increase in p53 levels within 6 h that declined by 24 h, whereas high doses caused a slow p53 accumulation. p21 levels were elevated after low- but not high-dose UVC exposure. As noted by those authors [41], many of these findings are consistent with earlier studies from other groups [46,47,48], including ours [49]. A similarly marked dose dependence of the cellular response to DNA damage was seen in MCF7 human breast cancer cells, where a high (40 J/m^2^) but not a low (2 J/m^2^) dose of UVC caused MDM4 translocation to the mitochondria, where it exerted a novel activity by blocking BCL-2 and promoting apoptosis [42]. This MDM4-mediated mitochondrial proapoptotic activity also appeared to influence human tumour-cell susceptibility to cisplatin [42]. We will return to the subject of nonlinear dose relationships in the cytotoxic/cytostatic responses of tumour and normal cells to cisplatin and UVC in Section 4.

The p53 and p21 proteins and their regulation are therefore central to decisions that relate to the mode of loss of cellular CF ability (“proliferative cell death”)—be that via a bona fide cell-death pathway such as apoptosis or via sustained proliferation arrest (cytostasis/dormancy)—as expanded below in Section 4.2. Given that p53 is the most commonly mutated gene in human cancer, many of these responses are likely to be perverted in cancer cell types with p53 defects, a scenario that we will also revisit in Section 4.

## 3. p53 Is an Early Stress Level-Dependent Rheostat in the Response to Electrophilic and/or Oxidative Agents

In addition to its central role in the DDR, p53 also plays an important early cytoprotective role after low-moderate levels of genotoxic/oxidative stress by promoting the elimination of potentially harmful electrophilic and prooxidant species before they can cause injury to the genome and other key cellular targets. It does this in part by upregulating genes with p53 response elements in their promoters that encode key cytoprotective proteins such as TIGAR (p53-induced glycolysis and apoptotic regulator) and GLS2 (glutaminase 2) [39]. TIGAR exerts its cytoprotective function in part by redirecting cellular metabolism from glycolysis (prooxidant) to the PPC, which generates the key cellular reductant NADPH and which, as noted above, can be an important factor in the activity of electrophilic drugs such as cisplatin. GLS2 is critical for the synthesis of GSH and thus for the cellular defense against electrophiles and prooxidants. In marked contrast, after high levels of genotoxic/oxidative stress, p53 undergoes a functional inversion and instead triggers a variety of mechanisms that lead to elevated levels of prooxidants and to cell death or cytostasis [50,51,52,53]. Again, this prooxidant function of p53 appears to have multiple components. For example, it can transcriptionally transactivate a number of genes/proteins with known connections to prooxidant and/or proapoptotic activity [51].

Another dose-dependent cytoprotective response orchestrated largely by p53 involves the stress-inducible Nrf2 transcription factor that can activate numerous target genes that encode proteins with anti-electrophile/antioxidant activities [54]. These include several known modulators of cisplatin activity, such as γ-glutamylcysteine synthetase (which is important in GSH synthesis), metallothioneins, Trx, TrxR, and key components of the PPC such as glucose-6-phosphate dehydrogenase and phosphogluconate dehydrogenase. Exposing cells to low-to-moderate levels of various prooxidants such as hydrogen peroxide or oridonin or of the anticancer drug etoposide was accompanied by elevated cellular Nrf2 protein levels, a response that was accentuated by the concomitantly activated *WT* p53 function through its transcriptional target p21 [55]. However, severe levels of oxidative and/or genotoxic stress and of p53 activation resulted in the opposite effect, i.e., a dose- and p53-dependent decrease in the levels of Nrf2 [55]. The presumed purpose of suppressing Nrf2 signaling in concert with the activation of prooxidant responses in highly damaged cells after severe or prolonged stress is to generate a more potent signal for either a cytotoxic or cytostatic response. To our knowledge, no studies have specifically characterized the impact of low versus high concentrations of cisplatin on cellular Nrf2 levels. Nrf2 does regulate several of the genes mentioned in Section 1 that encode drug-detoxifying enzymes and drug transporter/exporter proteins implicated in the cellular response to cisplatin, such as the MRPs and GSTs in addition to ABCF2 [56], another member of the ATP-binding cassette (ABC) transporter superfamily that includes the MRP1/2 and MDR1 proteins. Nrf2 has also been reported to activate the antiapoptotic BCL-2 gene [57,58], an activity whose reversal at severe stress levels could reinforce establishment of the prooxidant/proapoptotic state.

Although UVC is regarded as a poor activator of the Nrf2 response in some cell types [59], exposure of HCT116 colon carcinoma cells to UVC (20 J/m^2^) did cause a marked activation of Nrf2 and transcription of its downstream target heme oxygenase-1 (HO-1), likely by promoting the generation of ROS [60]. This pathway might thus play a role in the cellular response to UVC in some circumstances.

Another set of p53-regulated genes that have been associated with establishing a cellular prooxidant/proapoptotic state under severe/prolonged stress conditions are the p53-inducible PIG/TP53I genes [51,61]. We will return to these high-stress proapoptotic signals later, but first, we will consider in some detail the responses of normal and cancer cells to UVC, cisplatin and its analogs such as oxaliplatin and carboplatin at the cellular level.

## 4. The Apoptotic “Threshold” and Cell-Fate Decisions

It is apparent from the above discussion that the dose-response relationships for many of the key molecular events that underlie the cellular response to genotoxic/oxidative stress are nonlinear. These molecular events and their downstream signaling outputs regulate both the type and extent of cell fate, notably with respect to cytotoxic (e.g., apoptosis) versus cytostatic/dormancy outcomes. A clear illustration of nonlinear dose-response curves for these later cell-fate outcomes is the frequent observation of a dose “threshold” for the activation of apoptotic responses by many DNA-damaging agents, including UVC [41,49,62,63,64,65]. We will begin by considering several datasets obtained with UVC because of the above-noted much lower impact of potential confounding variables across cell lines and individual studies with this agent.

### 4.1. Apoptotic Threshold and Alternative Cell Fates Following Exposure of Normal and Malignant Human Cells to UVC

**Figure 2:**
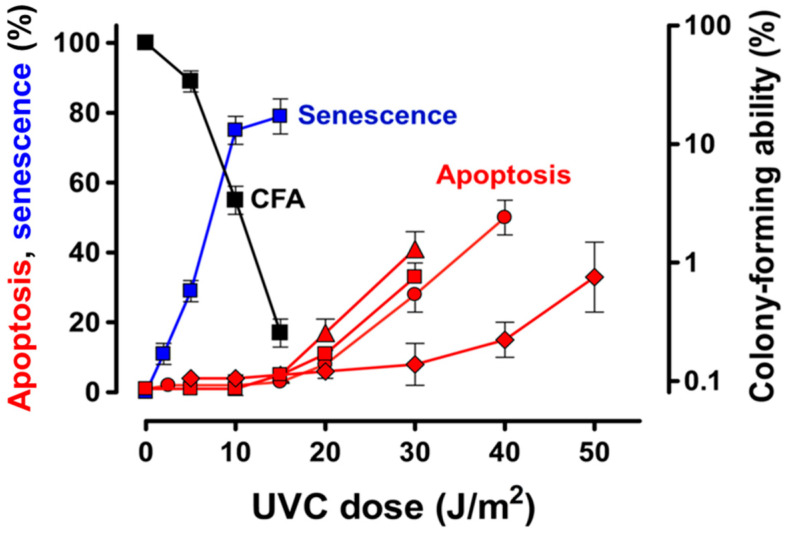
Dose-response curves for loss of colony-forming ability, apoptosis and therapy-induced cell senescence (TCS) in normal human fibroblasts following exposure to increasing doses of ultraviolet light-C (UVC) (254 nm): (■) Colony-forming assay (CFA) data for GM38 cells [49]; (■) UVC-induced TCS in GM38 cells at 7 days after UVC exposure based on the number of cells that retained viability, exhibited flattened and highly enlarged morphology, and were positive for senescence-associated β-galactosidase activity [62]; (●) UVC-induced apoptosis in 2525T cells based on the flow cytometric sub-diploid DNA content assay [49]; (■) UVC-induced apoptosis in GM38 cells based on flow cytometric determination of Annexin V-positive (phosphatidylserine externalized) cells [62]; (♦) UVC-induced apoptosis in primary 293T fibroblasts based on the Annexin V assay [65]; and (▲) UVC-induced apoptosis in AG1522 fibroblasts based on sub-diploid DNA content [64]. All apoptosis data were assessed at 72 h after UVC exposure.

Several early reports indicated that 254-nm UVC exposures induced apoptosis in various cell types; however, these studies invariably used high (supralethal) doses of UVC, e.g., 20–50 J/m^2^ (reviewed in [3]), presumably in anticipation of generating a suitably high signal-to-noise ratio, as noted above. Studies from our laboratory using non-transformed normal human fibroblasts with *WT* p53 [49,62], which are included in Figure 2, confirmed that significant levels of apoptosis occurred after supralethal doses of UVC. The apoptosis seen after such high doses was associated with a marked (approximately 20 fold) upregulation of p53 but with inhibition of induction of the antiapoptotic p21 protein that was seen after lower doses (see below). However, exposure to low-moderate doses of UVC covering the range where progressive loss of clonogenic potential was seen in the CF assay (i.e., up to 15 J/m^2^) caused no detectable apoptosis. Thus, there was a clear apoptotic threshold that coincided with the point where the clonogenic potential of the cells was greatly reduced (to ˂0.1% of the control level). Clement et al. [65] also observed minimal induction of apoptosis in non-transformed normal human fibroblasts exposed to ˂20 J/m^2^ of UVC, but again, exposure to high/supralethal doses resulted in significant levels of apoptosis, reaching approximately 30% at 50 J/m^2^ (Figure 2). Similar observations were reported by Latonen et al. [41]; exposure of human diploid fibroblasts to a low dose of UVC (10 J/m^2^), which would result in approximately 50% loss of clonogenic potential in the CF assay, primarily invoked a cell cycle arrest, whereas a high/supralethal dose (50 J/m^2^) resulted in approximately 60% of the cells undergoing apoptosis within 48 h based on sub-diploid DNA content, nuclear morphology and cleavage of poly (ADP-ribose) polymerase. Again, the selective apoptosis seen after high-dose UVC was associated with strong induction of p53 but not of p21. Studies by Stubbert et al. [64] indicated a similarly distinct threshold at approximately 20 J/m^2^ for UVC-induced apoptosis in three normal human fibroblast strains (GM38, NF and AG1522); their data with AG1522 cells for the endpoint of sub-diploid DNA content are included in Figure 2 but were confirmed using propidium iodide staining and caspase-3 activity assays.

An obvious question here is, if cells exposed to survival-curve range doses of UVC (i.e., ≤ 20 J/m^2^) are not undergoing significant apoptosis, then how are they losing their clonogenic potential? As anticipated in a pivotal commentary by Roninson et al. published almost 20 years ago that addressed this very question [66], exposure of fibroblasts to a range of low-moderate UVC doses where the progressive loss of clonogenic potential occurred resulted in a very different molecular and cellular response. Such doses caused a modest but significant (approximately 3 fold) upregulation of p53, prolonged nuclear accumulation of antiapoptotic p21, and activation of the senescence-like program of cytostasis/dormancy that we will refer to here as therapy-induced cell senescence (TCS) but which is also referred to by other names such as stress-induced premature senescence or accelerated senescence to distinguish it from replicative senescence [67], which is associated with telomere erosion. TCS cannot be simply defined using a single biomarker but rather is inferred from a combination of characteristics such as senescence-associated β-galactosidase (SA-β-gal) activity at pH 6 based on cleavage of the X-Gal substrate [68] in enlarged/flattened granular cells that have undergone a prolonged proliferation block (and thus fail to generate macroscopic colonies over the typical approximately 2-week duration of the CF assay) while retaining cell membrane integrity (i.e., viability) and metabolic activity [49,62,63]. In the GM38 fibroblast strain, for example, the TCS phenotype was seen in approximately 80% of the cells at 7 days after a 15 J/m^2^ UVC exposure, a dose that resulted in >99% loss of clonogenic potential (Figure 2). Minimal apoptosis (<2%) was seen at this dose level within 72 h ([49] and Figure 2). In short, as shown in Figure 2, the UVC dose-response for the loss of clonogenic potential in normal human fibroblast cultures closely parallels that for TCS but shows no relationship to apoptosis.

The responses to UVC described above do appear to extend beyond normal fibroblasts. For example, a similar threshold for UVC-induced apoptosis at approximately 20 J/m^2^ was seen in two p53-*WT* human solid tumour-derived cell lines, MCF7 (breast carcinoma) and HCT116 (colon carcinoma) [63].

### 4.2. Apoptotic Threshold and Alternative Cell Fates Following Treatment of Human Cancer Cell Lines with Cisplatin

As was noted in Section 1, most reviews on the effects of cisplatin on human solid cancer-derived cells favour a model in which loss of clonogenic potential is caused by unrepaired drug-induced DNA damage and inhibition of DNA replication triggering the DDR, which leads primarily to activation of the mitochondrial/intrinsic apoptotic pathway of cell death. However, as emphasized by Eastman [1], it is critical to perform such mechanistic studies at drug concentrations that reflect those that can be achieved clinically. For cisplatin, peak plasma concentrations in patients are approximately 10 μM, so anticipated tumour levels should be in the low-μM range. Critically, this is also the exact concentration range over which cisplatin-treated human tumour cell lines progressively lose their clonogenic potential in the CF assay or their proliferative capacity in the 3 day growth inhibition assay [69,70,71,72], suggesting that there may in fact be a connection between clinical efficacy and reduction of tumour-cell clonogenic potential.

An important survey of the early literature [69] indicated that the mean concentration of cisplatin used in preclinical studies to demonstrate apoptosis in various human cancer cell lines was in fact considerably higher (around 52 μM) than those that effectively eliminated the clonogenic/proliferative potential of the cells (i.e., ≤10 μM) and also noted that, when low-moderate, clinically-relevant concentrations of cisplatin had been tested, they had typically failed to induce significant apoptosis. Eastman [1] has cautioned that the many in vitro studies that used supralethal cisplatin concentrations—sometimes as high as 100 μM-probably have little relevance to how cisplatin exerts its therapeutic effects in cancer patients.

**Figure 3:**
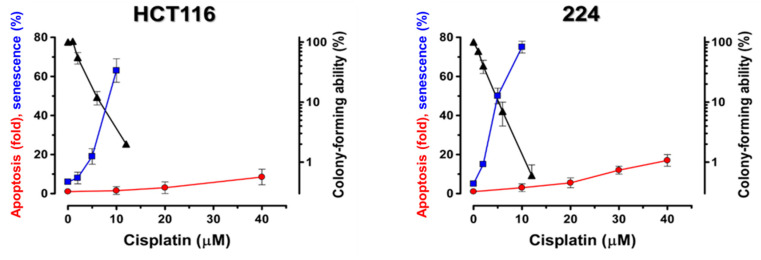
Dose-response curves for loss of colony-forming ability and for the induction of apoptosis and therapy-induced cell senescence (TCS) in HCT116 (colon carcinoma) and 224 (melanoma) p53-*WT* human tumour cells treated with increasing concentrations of cisplatin for 6 h. (▲) colony-forming ability assayed at 10 days posttreatment, (●) apoptosis based on caspase-3 activation assayed at 14 h posttreatment, and (■) TCS based on a combination of senescence-associated β-galactosidase activity and PKH2 staining assayed at 6 days posttreatment. Data from Berndtsson et al. [69].

**Figure 4:**
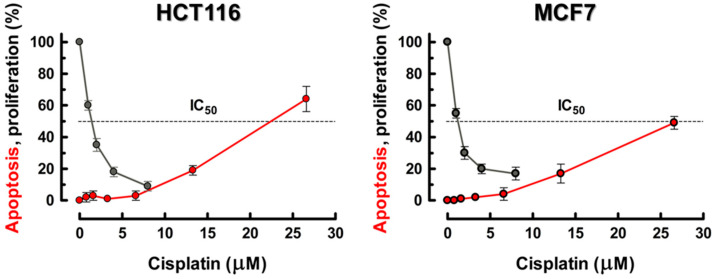
Dose-response curves for cisplatin-induced apoptosis and loss of proliferative potential in exponentially-growing cultures of the MCF7 and HCT116 p53-*WT* human cancer cell lines. (●) apoptosis data for cells exposed to increasing concentrations of cisplatin for 2 h. After 4 days, adherent and floating cells were combined, stained with Hoechst 33342 and propidium iodide and evaluated under a fluorescence microscope. Cells that had lost membrane integrity (i.e., propidium iodide-stained cells) as well as those exhibiting nuclear fragmentation were scored as apoptotic. From Mirzayans and Murray [63]. (●) Our unpublished data were obtained using the 3 day proliferation inhibition assay which involves direct cell counting. The experiments were performed as described [72] except that cells were incubated with cisplatin for 24 h followed by incubation in drug-free medium for 48 h.

As with UVC, described above, it is apparent from selected studies that used a broad range of cisplatin and oxaliplatin concentrations that span the low/clinically-relevant all the way through to high/supralethal ranges and that used assays that report on numbers of cells affected (rather than cell population-averaged assays) that there is again a distinct apoptotic threshold for these drugs, typically at approximately 10 μM. The necessity for using much higher concentrations of cisplatin or oxaliplatin to demonstrate cancer-cell apoptosis versus loss of clonogenic potential has been reported by many laboratories (e.g., [73,74,75,76,77]). Berndtsson et al. [69] confirmed the threshold nature of the apoptotic response in p53-*WT* HCT116 colon carcinoma and 224 melanoma cells exposed to cisplatin for 6 h, with significant levels of apoptosis only being seen after much higher cisplatin concentrations (threshold at approximately 10 μM) than those used in the CF assay, as shown in Figure 3. Figure 4 shows similar data from our laboratory with p53-*WT* MCF7 and HCT116 cancer cells after treatment with cisplatin for 2 h [63]. Both cell lines exhibited significant levels of apoptosis, but only after concentrations above a threshold at approximately 10 μM cisplatin, i.e., again above the range where the companion 3-day proliferation inhibition experiments were done. However, there are exceptions to this behavior, and some solid tumour-derived cell lines do exhibit manifestations of apoptosis after lower concentrations of cisplatin (e.g., [78,79]). From the future cancer therapy perspective, this is an important consideration that we will return to in Section 5.

Again, as with UVC, the paucity of early (≤3 days) apoptosis seen in many solid tumour-derived cell lines after treatment with clinically-relevant concentrations of cisplatin, i.e., ≤10 μM, poses an obvious question: what *is* the fate of these cells that results in their loss of clonogenic potential in the CF assay? Many studies over the last 20 years have shown that treating a variety of human solid tumour-derived cell lines with moderate, clinically-relevant concentrations of cisplatin and oxaliplatin causes only minimal cell death/apoptosis but they largely invoke a cytostatic/dormancy response characterized by a sustained proliferation arrest and cell enlargement [2,14,66,69,72,73,74,75,77,80,81,82,83,84].

In p53-*WT* cancer cells, cytostasis/dormancy is typically associated with characteristics similar to those described in Section 4.1 for normal fibroblasts exposed to UVC and is attributed to implementation of the senescence-like TCS program. For example, the above-cited paper by Berndtsson et al. [69], which showed a clear apoptotic threshold at approximately 10 μM, concluded that, while biologically/clinically-relevant concentrations of cisplatin (i.e., ≤20 μM) did not induce apoptosis, they did cause p53-*WT* HCT116 colon carcinoma and 224 melanoma cells to undergo an extensive and sustained growth arrest that was associated with SA-β-gal activity and an approximately 3-fold increase in p53 levels. This TCS response occurred over the same concentration range (approximately 110 μM), where the loss of cellular clonogenic potential was apparent in the CF assay (Figure 3). Whereas these moderate concentrations of cisplatin invoked extensive TCS in p53-*WT* HCT116 cells, this effect was greatly reduced in their homozygous p53-knockout counterparts [69]. A similar effect was seen in p53-*WT* H460 non-small-cell lung cancer cells [84], where treatment with a low concentration (7.9 μM) of another platinum-based anticancer drug, carboplatin, that caused approximately 50% loss of clonogenic potential in the CF assay caused very little apoptosis but a significant proportion of the cells displayed the enlarged/flattened morphology and SA-β-gal-positivity characteristic of TCS as well as increased levels of p53 and p21. We have made similar observations with p53-*WT* A549 lung carcinoma, HCT116 colon carcinoma and MCF7 breast cancer cells, where exposure to low (i.e., CF-assay range) concentrations of cisplatin (≤10 μM for 72 h) primarily triggered a cytostatic response with characteristics consistent with activation of the TCS program (unpublished data cited in [30,72]).

**Figure 5:**
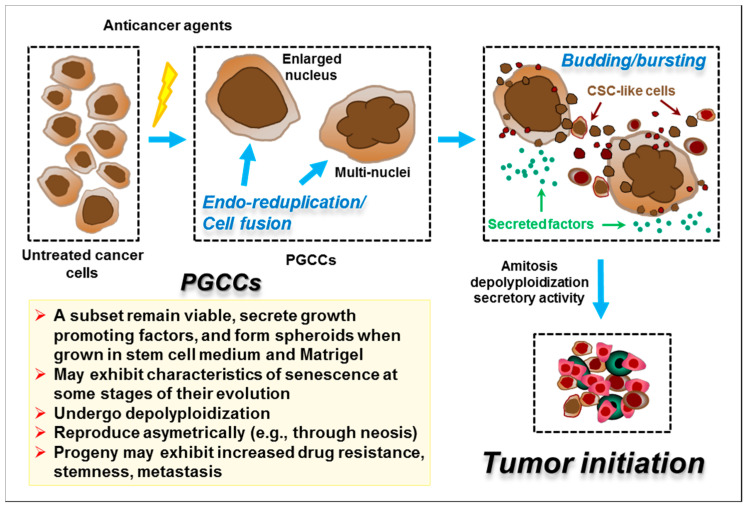
Cartoon illustrating the generation and fate of polyploid giant cancer cells (PGCCs). Cancer cells (top, left) stressed by factors such as anticancer agents or hypoxia can undergo a complex series of adaptations, including endoreduplication and cell fusion, that result in the emergence of PGCCs with either a highly enlarged nucleus or multiple nuclei. PGCCs may contribute to tumour repopulation following cancer therapy by at least three mechanisms: (i) depolyploidization through undergoing a complex genome reduction process, mediated by key regulators of mitosis, meiosis and self-renewal, ultimately giving rise to para-diploid progeny (i.e., containing a near-diploid number of chromosomes) that exhibit recovery of proliferative capability; (ii) depolyploidization by amitotic processes (e.g., neosis), involving budding and bursting (nuclear fragmentation) similar to prokaryotes and unicellular eukaryotes, to generate tumour initiating cells with cancer stem cell (CSC)-like properties; and (iii) secretion of factors that create a permissive tissue microenvironment for tumour growth and progression. Some features of PGCCs are listed in the left lower box. For details, please consult [85,87,88].

Exposure of solid tumour-derived cell lines that express mutant or no p53 to clinically-relevant concentrations of cisplatin and oxaliplatin also invokes a cytostatic/dormancy response. This response often involves the generation of what we will refer to here as “polyploid giant cancer cells” (PGCCs), as adopted in recent reviews of this topic [85,86]. Figure 5 illustrates the formation and fate of PGCCs after treatment of cancer cells with anticancer agents.

PGCCs are characterized by long-term proliferation arrest, a high degree of cell enlargement (often 10–20 fold but sometimes ≥100 fold), extensive polyploidy (including cells with a single enlarged nucleus or with multiple nuclei), retention of cell membrane integrity/viability, adherence to the tissue culture dish for long times (weeks) posttreatment and the ability to metabolize the probe MTT (3-(4,5-dimethylthiazol-2-yl)-2,5-diphenyltetrazolium bromide)—often to a significantly *greater* extent than untreated cells on a per-cell basis; they also exhibit secretory activity and have the potential to generate para-diploid progeny that can acquire the expression of cancer stem cell markers and contribute to drug resistance and metastasis [30,70,71,85,86,87,88,89,90,91,92,93].

Low levels of PGCCs have been detected in tumour samples from cancer patients at the time of diagnosis, including in many cancer types that are often treated with platinum-based drugs, such as those of the lung, breast, ovary, colon and bladder; indeed, PGCCs may represent a cancer “keystone” species that play a critical role in disease progression [86]. The levels of PGCCs can increase markedly following treatment with chemotherapeutic drugs [85,86]. Thus, treatment of the p53-*WT* PROb rat colon carcinoma cell line with clinically-relevant concentrations of cisplatin (5–10 μM, 3-h exposure) caused only a minor increase in apoptosis biomarkers within 3 days; rather, such exposures induced a cytostatic/giant-cell response in the tumour cells both in vitro and in vivo in a syngeneic subcutaneous tumour model [94]. Many of these giant cells exhibited SA-β-gal positivity, a point that we will return to below. Similar responses have been seen in human solid tumour-derived cell lines. For example, we observed that treating cultures of MDA-MB-231 breast carcinoma cells (which have a gain-of-function p53 mutation and which contain a subpopulation of giant cells even prior to drug exposure [89]), with moderate (i.e., CF-assay range) concentrations of cisplatin (≤ 10 μM for 72 h) again primarily triggered a cytostatic rather than cytotoxic response, with > 50% of the cells exhibiting prominent PGCC characteristics [72]. Exposing MDA-MB-231 cells to moderate concentrations of oxaliplatin (≤10 μM for 72 h) resulted in a similar cytostatic response that largely reflected the formation of PGCCs [70]. Similar responses to cisplatin were seen in other cancer cell lines expressing mutant p53 (SUM159) or no p53 (HCT116 p53-knockout) (R. Mirzayans, unpublished data cited in [30]).

Our understanding of the biology of chemotherapy-induced cancer-cell dormancy is rapidly emerging, but an in-depth discussion of this topic is beyond the scope of the present article. However, there are several caveats and questions that clearly need to be addressed. First, some researchers have investigated cytostatic responses to anticancer drugs primarily from the perspective of their contribution to the loss of cellular clonogenic potential in the CF assay (and thus potentially to early tumour responses) and have therefore focused on events occurring at early times, typically between 3 days and 2 weeks posttreatment. Such studies tend to infer that TCS and PGCC formation represent distinct cytostatic outcomes, with the former being largely the purview of p53-*WT* cells and with loss of *WT* p53 function seemingly being permissive for PGCC formation. These outcomes do, however, have significant elements in common, such as a high degree of enlargement of adherent cells that retain cell membrane integrity, secretory activity and the ability to metabolize MTT [30,70,71]. Unfortunately, there is no definitive individual biomarker or assay for either manifestation of cytostasis, and the incidence and uniqueness of these events has to be inferred from some combination of microscopic observations of cell morphology combined with endpoint-selective biomarkers such as SA-β-gal activity for TCS. The original paper that described the use of the SA-β-gal test as a biomarker for TCS [68] actually cautioned against its use in isolation. Indeed, many studies indicate that some PGCCs exhibit hallmarks of senescence, such as SA-β-gal positivity, at least at some stages of their evolution [95]. Furthermore, studies that have examined the longer-term fate of dormant cells indicate a significant potential for bidirectional interconversion between these phenotypes [3,71,87,93,96,97,98].

A second caveat is that we have used the term “dormancy” here to describe cells that undergo a prolonged growth arrest following exposure to DNA-damaging agents. In reality, these cells are anything but static. Indeed, they exhibit considerable ongoing metabolic activity related in part to the evolution of polyploidy/senescence and subsequent depolyploidization, which can eventually lead to the emergence of para-diploid progenitor cells through a variety of mechanisms; these normal-sized progeny are highly proliferative and are variously associated with the induction of epithelial to mesenchymal transition (EMT) and acquisition of stem-cell markers [85,86,87,88,93,95,99,100,101,102,103,104].

A third caveat relates to a consistent feature of our abovementioned studies using MDA-MB-231 cultures and noted by others (e.g., see [96] and references therein) in which individual cells within a drug-treated culture could undergo very different fates such that cells exhibiting manifestations of either TCS, PGCC formation or apoptosis were apparent in the same tissue culture dish. For this reason, methodologies that allow longitudinal monitoring of individual cells following exposure to a chemotherapeutic drug (rather than population-averaged readouts) will provide critical insight into the complexities of the temporal evolution of each response as well as their interrelationships. They may also indicate whether these different responses occur in the same or different cells. Such methodologies are particularly important for studying the evolutionary lifestyles of drug-treated cancer cells in relation to their potential long-term impact on tumour control (e.g., relapse, emergence of drug resistance and metastasis). One such study by Rohnalter et al. [92] used such longitudinal observation methods to follow the fate of SKOV3 ovarian cancer cells (which are p53-null) following treatment with carboplatin. At both the phenotypic and molecular levels, these authors observed a highly dynamic profile for individual cells over a 30-day period that included marked temporal changes in cell size, ploidy, SA-β-gal activity and senescent-like morphology as well as in p21 expression in relation to 5-bromo-2′-deoxyuridine incorporation into DNA; it also encompassed interchangeable lifestyle opportunities that could ultimately lead to the emergence of para-diploid, proliferating and potentially drug-resistant progeny.

### 4.3. What Drives the High-Dose Apoptosis Seen after UVC and Cisplatin/Oxaliplatin Treatment

The mechanism by which high/supralethal doses of UVC ≥20 J/m^2^ trigger a strong proapoptotic signal has primarily been equated to the fact that such exposures induce high levels of bulky/distorting DNA photoproducts that pose a physical block to transcription-coupled NER (e.g., [105]). This blockage impedes the transcriptional activation of stress-induced genes such as the p53-regulated p21 gene (the encoded protein of which is antiapoptotic) and of negative regulators of p53 (such as MDM2 and MDM4) that would be seen after low-moderate doses, leading to a p53-high/p21-low state in which high unopposed levels of proapoptotic p53 lead to a potent proapoptotic signal and thus to extensive apoptosis [33,41,49,62]. A similar scenario was seen with the wild-type p53-induced phosphatase 1 (WIP1) which dephosphorylates many DDR proteins, including p53, Chk1 and MDM2 [106], and which is implicated in resetting the DDR and cell cycle in cells that have effectively implemented repair of DNA damage (e.g., [33]), but it is also antiapoptotic [106]. In MCF7 cells, low doses of UVC (≤10 J/m^2^) that activated cell cycle checkpoints induced WIP1 in a p53-dependent manner whereas high doses (20–100 J/m^2^) progressively suppressed WIP1 mRNA and protein levels in a p53-independent manner and triggered apoptosis [106]. Similar effects were seen in U20S (p53-*WT*) and Saos2 (p53-null) osteosarcoma cells [106]. Although the above-noted physical blockage of transcriptional transactivation is likely the major contributor to the suppression of WIP1 after high-dose UVC exposures, the authors of this study [106] noted that altered WIP1 protein stability and possibly microRNAs [107] may also play a role. As expected, p21 levels in that study were modulated in a similar manner to WIP1, being induced by low-dose exposures but suppressed after high doses [106].

The triggering of cellular proapoptotic responses after supralethal concentrations of cisplatin/oxaliplatin may also be driven in part by the same mechanism, i.e., by impaired transcription-coupled NER of drug-induced bulky DNA lesions leading to ineffective transcriptional activation of stress-responsive genes (e.g., [105]). However, cisplatin did not induce WIP1 in the above study [106], so there may be subtle differences between UVC and cisplatin in this regard.

Another mechanism that may contribute to enhanced proapoptotic signaling at high/supralethal levels of genotoxic stress that induce high levels of p53 involves the potential suppression of the Nrf2 pathway, as outlined in Section 3. Such a response has been described following exposure of cells to high concentrations of genotoxic agents such as hydrogen peroxide or the anticancer drug etoposide or simply by ectopic overexpression of p53 itself [55]. Suppressing the Nrf2 pathway leads to a failure to activate Nrf2-regulated pro-survival factors such as GSH, metallothionein, MRP1/2 and MDR1/p-glycoprotein, and the PPC-TrxR-Trx pathway. Although the high-stress suppression effect has not been specifically characterized for the platinum-based drugs, to our knowledge, many of these Nrf2-regulated proteins can significantly modify cisplatin activity. Indeed, Nrf2 expression has been associated with tumour-cell resistance to cisplatin [6] while Nrf2 inhibitors can sensitize various cancer cell lines and xenografts to cisplatin [4,108]. Intuitively, the impact of suppressing Nrf2 and its target proteins should be less important with physical (UVC) versus chemical (cisplatin/oxaliplatin) agents. Nonetheless, it might still contribute to high-stress apoptotic signaling with both agents because cisplatin/oxaliplatin [6,109] and to some extent UVC, either directly or via the release of inflammatory cytokines [110], cause significant generation of ROS that will be subject to regulation by the Nrf2 pathway. The above-noted block to the transcriptional transactivation of the p53-regulated p21 gene at very high levels of DNA damage might reinforce such an effect because p21 is an important stimulator of Nrf2 activation, as noted in Section 3.

The generation of proapoptotic signals after very high levels of genotoxic stress may also be driven in part by the p53-inducible PIG/TP53I proteins, as outlined in Section 3. Several of these proteins have been implicated in establishing a prooxidant/proapoptotic phenotype in cells experiencing supra-physiologically high levels of p53 [51]. Although that original observation was based on ectopic overexpression of p53 [51], this phenomenon was anticipated to extend to cells experiencing extensive p53 induction/activation as a result of high/supralethal levels of genotoxic stress [50]. We are not aware of any studies that have directly addressed such a role for the PIG/TP53I proteins in the response of cells to high levels of UVC or cisplatin/oxaliplatin where extensive apoptosis is seen. However, a potential role for individual members of this group has been implicated in some cases. For example, PIG1 (galectin-7), which can stimulate superoxide generation as well as regulate apoptosis through jun N-terminal kinase (JNK) activation and mitochondrial cytochrome c release [111,112], was induced by cisplatin in urothelial cancer cell lines with *WT* p53 but not with mutant p53 [113]. Furthermore, transfecting p53-mutant cancer cells with galectin-7 sensitized them to cisplatin by increasing intracellular levels of ROS and by activating the JNK pathway [113]. We should note, however, that the suppression of the transcriptional transactivation of p53-regulated genes such as p21 and WIP1 after supralethal genotoxic exposures as a result of an impaired repair of bulky DNA lesions (summarized earlier in this section) may play a role with UVC or platinum-based drugs by reducing the extent of PIG/TP53I activation and therefore may diminish any impact that they might have on proapoptotic signaling.

As expected, considering that the very subject of this section is the biphasic threshold-dependent activation of the apoptotic machinery by UVC and cisplatin, numerous studies have shown that various interactions between pro- and antiapoptotic mitochondrial proteins are selectively altered after supralethal UVC and cisplatin exposures [41,42,114]. The intriguing possibility has been considered [30] that apoptosis induced in solid tumour-derived cell lines by high/supralethal concentrations of cisplatin and oxaliplatin may in fact be primarily caused by drug-induced damage to mitochondria rather than to genomic DNA [14,69,75]. Indeed, Berndtsson et al. [69] suggested that whereas the TCS response observed in p53-*WT* human cancer cells exposed to low biologically/clinically-relevant concentrations of cisplatin may be triggered primarily by DNA damage, the apoptosis seen after high/supralethal concentrations of the drug (Figure 3 and Figure 4) is perhaps an “off-target” response to cytoplasmic damage/ROS generation, possibly triggered by cisplatin-protein adducts. If this model is correct, then the apoptosis seen after supralethal insults should be independent of the cells’ DNA repair status whereas the low-dose TCS response should be greatly influenced by this parameter. The same caveat should apply to any component of apoptosis triggered by ER stress and the related UPR independently of DNA damage; this response does appear to engage at relatively low stress levels [11,12], although oxidative damage to proteins can trigger both cytoprotective and apoptotic pathways depending on the extent of damage [39].

## 5. Identification of Drugs That Transition Dormant or Potentially Dormant Cancer Cells to a Bona Fide Cytotoxic Pathway

Neither manifestation of the cytostasis/dormancy seen in solid tumour-derived cancer cell lines after clinically-relevant exposures to anticancer agents such as cisplatin/oxaliplatin represents a cell “death” pathway per se. While triggering dormancy responses in the clinical setting might provide a significant progression-free interval, both the TCS and PGCC states can develop instability, providing a route to cell survival; a subpopulation of unstable cells that generate proliferating progeny at late times and may acquire stem-cell characteristics has the potential to cause disease relapse and therapy resistance [26,30,70,80,85,93,94,96,104,115,116,117,118,119]. The threat posed to patients by treatment-induced dormant cells of all forms is exacerbated by their secretory activity that drives their progression towards recurrence and metastasis as well as potentially mediates resistance to later cycles of chemotherapy for which the effects will be superimposed on a tumour environment in which many of the cells will already be in a dormant state and thus actively engaged in such secretory activity. For cells that have undergone TCS, this activity is known as the senescence-associated secretory phenotype or “SASP” [120]. The risk to patients from PGCCs is starkly apparent from their highly tumourigenic nature: thus, PGCCs isolated from a murine fibrosarcoma [121] or from various human cancer cell lines, including MDA-MB-231 breast carcinoma [85], were capable of generating tumours with high efficiency when injected into a host mouse.

The initial period of tumour dormancy provided by platinum-based chemotherapy drugs may nonetheless provide a window of opportunity to intervene before the impact of instability and secretory activity can manifest. One strategy would be to selectively target these dormant but viable cancer cells for destruction using adjuvant therapies that can pharmacologically transition them into a bona fide cell-death pathway such as apoptosis from which they will not return. There is in fact a precedent for this strategy dating back to 1956 when Puck and Marcus [90] reported that exposure of cultured HeLa cervical carcinoma cells (which lack functional p53 as a result of their infection with HPV-18) to X-ray doses typical of those used in the CF assay resulted in the extensive formation of dormant giant cells. Remarkably, these dormant/giant cells were highly susceptible to destruction by Newcastle disease virus [90]. The many current oncolytic viruses may thus provide one potential source of useful adjuvant therapeutics for eradicating dormant cancer cells generated by anticancer drugs such as cisplatin.

Several strategies have shown promise for transitioning the senescence-like TCS phenotype into a cell death/apoptotic pathway. This approach has recently become a subject of major interest in the cancer therapy domain and has seen the adoption of the monikers “senolytics” [122] or “senotherapy” [123] to describe such strategies. It is important to remember that, as discussed in Section 4.2, most solid tumour-derived cell lines do appear to be capable of activating apoptosis after exposure to platinum-based drugs at some concentration level, so their apoptotic pathways must be functional and therefore capable of being triggered by suitable approaches. Candidate targets for inhibition using senolytics would include proteins that maintain the senescence-like state, such as the positive regulatory loop involving p21 and ATM. Indeed, ATM small-molecule inhibitors as well as p21 antisense oligonucleotides triggered apoptosis in cultures of the A549, MCF7 and HCT116 cancer cell lines (all p53-*WT*/p16^−^) that had previously undergone doxorubicin-induced TCS [40]. Another strategy is to inhibit antiapoptotic proteins and thereby lower the cellular apoptotic threshold. In one such study [124], targeting survivin, a member of the inhibitor of apoptosis protein (IAP) family, using HIV-1 TAT peptides to disrupt the phosphorylation of survivin by cyclin-dependent kinase 1 abrogated the TCS response seen in H1299 lung cancer cells that had been treated with the anticancer drug camptothecin and instead triggered an apoptotic response.

Inhibitors of antiapoptotic proteins could also be efficacious in the context of lowering the apoptotic threshold of tumour cells *prior* to the delivery of the genotoxic therapeutic drug, such that a tumour cell that would otherwise have primarily undergone cisplatin-induced TCS might now directly enter an apoptosis pathway. Potential targets include antiapoptotic proteins such as BCL-2 or BCL-XL (B-cell lymphoma-extra large) [116]. Although we are not aware of any studies of this type with the platinum-based drugs specifically, Hsu et al. [125] recently reported that treatment with the BCL-XL/-2/-w small-molecule inhibitor ABT-263 (Navitoclax) caused a loss of viability in A549 cells that had previously undergone doxorubicin-induced TCS. Other targets for which commercially available inhibitors are available include the mammalian target of rapamycin (mTOR), a key regulator of cell metabolism, autophagy, apoptosis, and phospholipase D, which positively regulates mTOR. A different approach would be to target the altered metabolic features of TCS using drugs such as metformin and dichloroacetate [126].

It will be equally important to identify adjuvants that can transition cancer cells exhibiting PGCC characteristics following treatment with drugs such as cisplatin into a cell death/apoptosis pathway before they can exert their negative impact on long-term tumour control and metastasis. Targets of interest here again include the BCL-XL/BCL-2 antiapoptotic proteins on which PGCCs are exquisitely dependent; indeed, the BCL-XL/-2/-w inhibitor ABT-263 caused loss of viability of HCT116-derived PGCCs, especially when combined with Aurora B kinase inhibitors [127]. This approach has not been tested for activity against cisplatin- or oxaliplatin-induced PGCCs to our knowledge. However, some encouragement for evaluating this strategy can be derived from studies showing enhanced levels of apoptosis when cisplatin and inhibitors of antiapoptotic proteins are used in combination. For example, WEHI-539 (a BCL-XL-selective small molecule inhibitor) and ABT-263 potentiated apoptosis when combined with cisplatin (72 h concomitant treatment) in the DAOY medulloblastoma cell line [128]. ABT-263 also sensitized small cell lung cancer cells to cisplatin, an effect that was typically associated with enhanced apoptosis [129]. It also synergistically enhanced cisplatin-induced apoptosis in non-small-cell lung cancer cells regardless of their p53 status [130]. Another BCL-2 inhibitor, ABT-199 (Venetoclax), enhanced the effects of cisplatin and induced apoptosis in oral tongue squamous cell carcinoma cells [131]. There is also some evidence to suggest that such inhibitors can target PGCCs per se. Thus, ABT-263 or siRNA directed to BCL-XL triggered the rapid apoptotic demise of PGCCs in acute myeloid leukemia cell lines and primary bone marrow blasts that had been treated with polyploidy inducers [132], providing some cause for optimism that similar approaches might be effective in eradicating PGCCs that arise following treatment with anticancer drugs such as cisplatin. This potential needs to be addressed with some urgency. Other possible vulnerabilities/targets that might be exploited for triggering apoptosis of cisplatin-induced PGCCs include the following:

(i) the Aurora B kinase that functions in the attachment of the mitotic spindle to the centromere. Aurora B was reported to support the survival/proliferation of PGCCs formed following exposure of HeLa cervical carcinoma and Namalwa Burkitt’s lymphoma cell lines to ionizing radiation, whereas these cells underwent apoptosis in its absence [133].

(ii) the notch signaling pathway, which may play an important role in the genesis of PGCCs [87]; and

(iii) the acquired stemness of PGCCs as they evolve towards para-diploidy, which may represent an “Achilles heel” by conferring a susceptibility to drugs that can promote redifferentiation [87].

An alternative to targeting known candidate proteins would be to undertake agnostic high-throughput screening of libraries of chemical or biological inhibitors to identify molecules capable of redirecting these ultimately unstable pro-survival dormancy responses to apoptosis. A potentially useful approach for monitoring cytostatic to cytotoxic conversion in this setting would be to exploit the observation that virtually all persistent enlarged viable cancer cells—whether they display characteristics primarily of TCS or PGCCs—remain adherent to the tissue culture dish for extended periods [89]; on this basis, simply monitoring cell detachment may provide a powerful monitoring tool in this setting.

## 6. Concluding Remarks

The fundamental premise of using DNA-damaging drugs such as cisplatin and its analogs in cancer therapy is to eradicate or at least control the growth/progression of a tumour while not causing undue toxicity to patients. At the tumour-cell level, the clinically preferred response is clearly to activate a bona fide cell-death pathway such as apoptosis; indeed, few researchers would disagree with Dr. Don Coffey when he said that “the only good cancer cell is a dead cancer cell” (quoted in [134]). In an ideal world, cancer cells would readily undergo apoptosis following treatment with low/nontoxic dosages of therapeutic drugs, and indeed, such responses are typically seen in hematologic cancers and cell lines. Unfortunately, clinically-relevant doses of commonly used DNA-damaging anticancer drugs such as cisplatin and its analogs usually invoke minimal to modest apoptotic responses in solid tumour-derived cell lines, although this may be more prevalent in some cancer types such as those of germ-cell origin [23]. Apoptosis does seem to be a default response of cancer cells to high/supralethal concentrations of these drugs, as discussed in Section 4.2; however, this phenomenon has little clinical relevance other than to suggest that the pathways that lead to apoptosis must be intact/functional in these cells and are therefore potentially susceptible to activation using alternative strategies, as outlined in Section 5.

In many cases, the primary fate of solid tumour-derived cell lines following treatment with clinically-relevant concentrations of cisplatin and its analogs is cytostasis/dormancy. Dormant cells primarily exhibit characteristics of either TCS or PGCCs depending on a variety of factors but notably cellular p53 status. While dormant cancer cells may contribute to an extended interval of progression-free survival in the clinic, the possibility that they might develop instability and regenerate proliferative progeny at later times could ultimately contribute to treatment failure as well as disease dissemination. As pointed out herein and previously [103], it is important to note that the key roles played by cytostatic/dormant cancer cells in tumourigenesis, metastasis and disease relapse after conventional cancer treatments have been well documented for different biological systems (e.g., tumour-derived cell lines, animal models and specimens from cancer patients) and for various types of solid tumours, including ovarian cancer, prostate cancer, brain cancer, renal cancer, colon cancer and breast cancer, including the most aggressive form (triple negative) for which chemotherapy remains the cornerstone therapeutic (reviewed in [103]).

If robust biomarkers for a “permanent” state of therapy-induced dormancy for a given patient’s tumour could be identified, then it may be clinically appropriate to “leave well alone”. On the other hand, if other biomarkers can reliably identify tumours that have a high probability of relapse because of their propensity to generate unstable dormant cells that can regenerate proliferative progeny, then the period of dormancy could still offer the prospect of intervention with an adjuvant therapeutic that could transition the initial dormancy response into apoptosis. In other words, achieving the “Holy Grail” of tumour apoptosis just may require more effort—notably via the 2-stage use of adjuvants to trigger the death of cancer cells that have initially undergone cytostasis/dormancy in response to the primary therapy (Figure 1, green arrow). Alternatively, promoting apoptosis over dormancy up-front by combining either neoadjuvant or concomitant proapoptotic drugs (e.g., ABT-263) with cisplatin/oxaliplatin may provide a simpler approach that might not require risky biomarker-based decisions.

We strongly reiterate the cautionary note made by Eastman [1] that in vitro experiments done with very high concentrations of drugs such as cisplatin are very likely irrelevant and thus misinformative in the context of their clinical use. The widespread conviction that the efficacy of the platinum-based drugs for treating solid tumours is mediated largely by apoptotic cell death needs to be urgently reconsidered for clinically-relevant exposures based on these in vitro data. The magnitude of this problem is illustrated by the fact that a search of PubMed in June of 2020 for the terms *“cisplatin and apoptosis”* returned almost 11,000 hits compared to about 500 for *“cisplatin and senescence”* and 135 for *“cisplatin and polyploid/giant cell”*.

As far as extrapolating these concepts from cell lines to patients, a pilot study by Wu et al. [99,135] questioned whether chemotherapy-induced TCS might contribute significantly to treatment response. These authors evaluated 18 patients receiving neoadjuvant carboplatin/paclitaxel chemotherapy or radiochemotherapy for locally advanced non-small-cell lung cancer and observed a lower overall survival in patients whose tumours displayed high levels of TCS biomarkers. TCS in this small study cohort was therefore associated with poor treatment outcome and disease recurrence. A similar pilot study of 26 patients with malignant pleural mesothelioma receiving platinum-based neoadjuvant chemotherapy again suggested that the occurrence of tumour TCS was a negative prognostic factor [136]. Despite the recent surge of interest in PGCCs, no analogous clinical studies with chemotherapy patients have yet been undertaken to our knowledge.

The above-noted pilot clinical studies of TCS and chemotherapy, which of necessity used invasive analytical methodologies, were limited to an interrogation of surgical samples collected at the time of diagnostic biopsy and/or surgical resection. They clearly point the way to trying to understand the role of drug-induced cancer-cell dormancy in the clinical setting, but they also highlight the need for better biomarkers and, in particular, for noninvasive technologies that will allow the longitudinal monitoring of such responses, particularly using molecular imaging-based techniques. Again, there has been considerable progress in this regard in relation to imaging apoptosis in tumours [137] but the ability to noninvasively monitor cancer-cell dormancy remains elusive [138]. Without such capability, studies of these events will always be limited to taking a “snapshot” of what is in fact a highly dynamic process, as clearly illustrated in the abovementioned in vitro studies by Rohnalter et al. [92], who demonstrated the markedly time-dependent fate of individual cancer cells in cisplatin-treated cultures. Another level of complexity that will ultimately need to be addressed relates to ongoing research into the cellular dynamics of individual DDR components in single cells treated with DNA-damaging agents and how these multiple signals are integrated and translated into cell-fate decisions such as survival, death/apoptosis and dormancy/cytostasis [45,139,140,141].

Despite such complexities, as recently pointed out by White-Gilbertson and Voelkel-Johnson [142], the field of cancer cell dormancy (e.g., through formation of PGCCs) “is at an exciting moment, when bench-to-bedside research has the potential to make a difference in the lives of many cancer patients.” For those who are interested in further reading, we suggest seminal/recent papers on reversible cancer cell polyploidy and senescence [87,88,95,99,100,104,118,142,143] that were discussed herein as well as papers on related topics such as therapy-induced autophagy [95,144,145] and genome chaos/micronucleation [146,147,148] that were beyond the scope of the current review.

## Abbreviations

CF	Colony-forming
DDR	DNA damage response
UVC	ultraviolet light-C
ER	endoplasmic reticulum
UPR	unfolded protein response
OCT	organic cation transporter
MRP1/2	multidrug resistance-associated proteins 1 and 2
GSH	glutathione
MDR1	multidrug resistance protein 1
GST	GSH-S-transferase
PPC	pentose phosphate cycle
TrxR	thioredoxin reductase
Trx	thioredoxin
Nrf2	nuclear erythroid-like factor 2
ROS	reactive oxygen species
NER	nucleotide excision repair
FA	Fanconi anemia
CSC	cancer stem cells
*WT*	wild type
MDM2/4	murine double minute 2 and 4 homologues
ATM	ataxia telangiectasia-mutated
ATR	ataxia telangiectasia and Rad3-related
Chk1/2	checkpoint kinase 1 and 2
DSB	DNA double-strand break
BAX	BCL-2-associated X
PUMA	p53 up-regulated modulator of apoptosis
BCL-2	B-cell lymphoma 2
TIGAR	p53-induced glycolysis and apoptotic regulator
GLS2	glutaminase 2
ABC	ATP-binding cassette
HO-1	heme oxygenase-1
TCS	therapy-induced cell senescence
SA-β-gal	senescence-associated β-galactosidase
PGCC	polyploid giant cancer cell
MTT	3-(4,5-dimethylthiazol-2-yl)-2,5-diphenyl-tetrazolium bromide
EMT	epithelial to mesenchymal transition
WIP1	wild-type p53-induced phosphatase 1
JNK	jun N-terminal kinase
IAP	inhibitor of apoptosis protein
BCL-XL	B-cell lymphoma-extra large
mTOR	mammalian target of rapamycin
